# Children and Adolescent Obesity Associates with Pressure-Dependent and Age-Related Increase in Carotid and Femoral Arteries' Stiffness and Not in Brachial Artery, Indicative of Nonintrinsic Arterial Wall Alteration

**DOI:** 10.1155/2016/4916246

**Published:** 2016-03-15

**Authors:** Victoria García-Espinosa, Santiago Curcio, Juan Manuel Castro, Maite Arana, Gustavo Giachetto, Pedro Chiesa, Yanina Zócalo, Daniel Bia

**Affiliations:** ^1^Physiology Department, School of Medicine, Centro Universitario de Investigación, Innovación y Diagnóstico Arterial (CUiiDARTE), Republic University, General Flores 2125, 11800 Montevideo, Uruguay; ^2^Pediatric Clinic “C”, School of Medicine, Republic University, Pediatric Hospital Center Pereira Rossell, ASSE, Ministry of Public Health, Bulevar Artigas 1550, 11600 Montevideo, Uruguay; ^3^Pediatric Cardiology Department, Pediatric Hospital Center Pereira Rossell, ASSE, Ministry of Public Health, Bulevar Artigas 1550, 11600 Montevideo, Uruguay

## Abstract

*Aim*. To analyze if childhood obesity associates with changes in elastic, transitional, and/or muscular arteries' stiffness.* Methods*. 221 subjects (4–15 years, 92 females) were assigned to normal weight (NW, *n* = 137) or obesity (OB, *n* = 84) groups, considering their body mass index *z*-score. Age groups were defined: 4–8; 8–12; 12–15 years old. Carotid, femoral, and brachial artery local stiffness was determined through systodiastolic pressure-diameter and stress-strain relationships. To this end, arterial diameter and peripheral and aortic blood pressure (BP) levels and waveforms were recorded. Carotid-femoral, femoropedal, and carotid-radial pulse wave velocities were determined to evaluate aortic, lower-limb, and upper-limb regional arterial stiffness, respectively. Correlation analysis between stiffness parameters and BP was done.* Results*. Compared to NW, OB subjects showed higher peripheral and central BP and carotid and femoral stiffness, reaching statistical significance in subjects aged 12 and older. Arterial stiffness differences disappeared when levels were normalized for BP. There were no differences in intrinsic arterial wall stiffness (elastic modulus), BP stiffness relationships, and regional stiffness parameters.* Conclusion*. OB associates with BP-dependent and age-related increase in carotid and femoral (but not brachial) stiffness. Stiffness changes would not be explained by intrinsic arterial wall alterations but could be associated with the higher BP levels observed in obese children.

## 1. Introduction

The prevalence of childhood obesity has increased over the last decade and it has become a growing health problem. Obesity in childhood commonly leads to obesity in adulthood [1] and it has been demonstrated that childhood obesity predisposes to obesity-related disorders, clusters with other cardiovascular risk factors, and contributes to an increase in cardiovascular risk in adulthood [2, 3]. In relation with this, it is known that atherosclerosis begins early in life, especially in children exposed to cardiovascular risk factors. Then, childhood obesity could accelerate the atherosclerotic process and its association with other risk factors could lead to a premature development of atherosclerosis [4–6].

Noninvasive vascular evaluation has been recently included in recommendations for cardiovascular risk assessment and prevention in children and adolescents [5, 7, 8]. Related with this, atherosclerosis-related vascular damage could be early diagnosed or identified by the associated changes in arterial stiffness. In turn, this can be quantified by local (i.e., distensibility) and regional (i.e., pulse wave velocity) arterial stiffness parameters that give complimentary information, useful to assess vascular damage and cardiovascular risk [9–13]. Arterial stiffness mainly depends on the structural components of the arterial wall, on the vascular smooth muscle tone, and on blood pressure (BP) levels. If childhood obesity associates with changes in arterial stiffness levels and/or if changes in arterial stiffness levels can be adequately assessed by noninvasive techniques remains controversial. About this, several works showed different and/or opposite results (including increased, unchanged, or (even) reduced arterial stiffness level) in obese children [8]. Looking at the available data, it could be said that controversial results could be explained, at least partially, by differences in the arteries (arterial pathway) studied, applied techniques, and methodological approaches and/or by the lack of normalization for BP levels.

In this context, this work's aims were to evaluate whether childhood obesity associates with arterial stiffness changes and to determine (1) if the vascular impact of obesity would depend on the histological arterial type (elastic, transitional, or muscular) and/or arterial region (neck, thorax, abdomen, and upper or lower limb), (2) if the arterial stiffness changes would be associated with the increased BP found in obese children, and (3) if the vascular changes associated with obesity would depend on the children's age. To fulfil our aims, we studied arteries from different regions and histology using a noninvasive methodological approach; we calculated local and regional arterial stiffness and we applied an analysis that allowed arterial stiffness to become BP-independent.

## 2. Methods

All procedures agreed with the Declaration of Helsinki (1975 and reviewed in 1983). All studies were approved by the Institutional Ethics Committee of the Centro Hospitalario Pereira-Rossell. Written informed consent was obtained before the examination.

### 2.1. Subjects

CUiiDARTE Project is a Uruguayan Interdisciplinary University Program aimed at early diagnosis of arterial disease in children and adults. Children data were obtained from CUiiDARTE Database. We included 211 asymptomatic children (age range: 4–15 years old; 92 female). Children with chronic comorbidities, with cardioactive drugs use, or under treatment that could affect the cardiovascular system were excluded. Each child was assigned to one of two groups: normal weight (NW, *n* = 137) or obese (OB, *n* = 84). Obesity was defined taking into account the body mass index (BMI) *z*-score (*z*BMI) (see below).

### 2.2. Clinical Interview and Anthropometric Assessment

Before vascular evaluation, a clinical interview was performed in order to assess personal and familiar medical history and cardiovascular risk factors exposure. Children were classified as sedentary when their regular physical activity level was lower than a moderate intensity load [14]. Children were considered dyslipidemic and/or hypertensive if they had prior diagnosis of those conditions [15]. Hypertension was defined as systolic and/or diastolic BP greater than the 95th percentile for age, gender, and height on three or more separate occasions [15]. In addition, children with systolic and/or diastolic BP levels over 95th percentile for gender, age, and height (during the study) were considered to have hypertensive BP levels [15].

Children's height and weight were measured and the BMI was obtained by dividing body weight by height squared. Then, the *z*BMI was calculated as the difference between the BMI and the median BMI for age and gender, divided by a specific standard deviation according to age, gender, and BMI percentile [16]. Taking into account the *z*BMI, children were classified as NW (−1 ≥ *z*BMI ≤ +1) or OB (*z*BMI > +2).

### 2.3. Arterial Evaluation

Children and adolescents were studied in a temperature-controlled room (21°–23°C); they were instructed to lie in supine position for at least 15 minutes in order to achieve a steady hemodynamic state. Heart rate and peripheral (brachial) systolic (pSBP) and diastolic (pDBP) BP measurements were obtained at fixed intervals of 8–10 minutes (HEM-433INT; Omron Healthcare Inc., Illinois, USA). Peripheral pulse pressure (pPP, pPP = pSBP − pDBP) and mean blood pressure (MBP, MBP = pDBP + pPP/3) were calculated.

#### 2.3.1. Central Aortic Pressure

**Figure 1:**
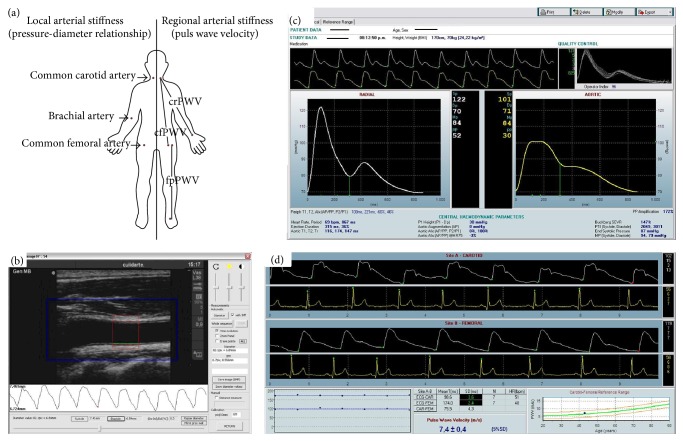
(a): Local (left) and regional (right) sites of arterial stiffness noninvasive evaluation. (b) Diameters border detection applied recreating common carotid artery diameter waveforms. (c) SphygmoCor Software, to determine central aortic blood pressure (levels and waveform) from radial pulse waveforms recordings. (d) SphygmoCor Software to determine arterial pulse wave velocity, from the consecutive recordings of proximal (i.e., carotid) and distal (i.e., femoral) pressure waveforms and electrocardiographic signals.

Radial pulse pressure waveforms were obtained using applanation tonometry (SphygmoCor 7.01, AtCor Medical, Sydney, Australia). The pDBP and MBP were used for radial pulse waveforms calibration. The central aortic BP was obtained using a validated generalized transfer function (SphygmoCor Software) applied to the acquired peripheral waves [Figure 1(c)]. Then, aortic (central) systolic, diastolic, and pulse pressure (cSBP, cDBP, and cPP, resp.) were quantified.

#### 2.3.2. B-Mode Ultrasonography

Left and right vertebral arteries, common carotid artery (CCA) and internal and external carotid arteries, common femoral artery (CFA), and left brachial arteries (BA) were analyzed to verify normal blood flow patterns. High-resolution B-mode ultrasonography (6–13 MHz linear transducer, M-Turbo, SonoSite Inc., 21919 30th Drive SE, Bothell, WA 98021, USA) was used to obtain sequences of images (videos) from transversal and longitudinal views of CCAs, CFAs, and left BA arteries, which were stored for offline analysis. Then, beat-to-beat diameter waveforms were obtained using border detection algorithm. Systolic (SD) and diastolic (DD) arterial diameters and the arterial wall intima-media thickness (posterior wall, end-diastolic value) were quantified as the average of at least 20 beats [Figure 1(b)] [11, 17].

#### 2.3.3. Local Arterial Stiffness

Cross-sectional arterial distensibility (AD) was quantified as AD = ((SD − DD)/DD)/PP. Central aortic PP was considered to quantify CCA AD, while pPP was used to quantify CFA and BA distensibility.

Childhood obesity is frequently associated with increased BP levels, which in turn could determine a transient BP-dependent increase in arterial stiffness due to a passive distension of the arterial wall. Then, to account for BP dependence of arterial stiffness, three complimentary data analyses were done. First, arterial stiffness was normalized for BP calculating the *β* Index (a widely used parameter): *β* = ln⁡(SBP/DBP)/((SD − DD)/DD). This parameter takes into consideration the theoretical exponential relation between BP and arterial diameter.

Second, the intrinsic (with independence of BP and geometry) stiffness of the arterial wall was evaluated by means of the arterial wall elastic modulus (EM). EM conceives the vessel as a hollow structure and provides information about the wall artery material regardless of its geometry and/or size [17, 18]. To this end, the left and right CCA and CFA wall thickness (intima-media thickness), strain (*ε*), and circumferential stress (*σ*) were calculated as previously reported [17]. Systolic and diastolic *σ* (Lamé's equation) were calculated as 0.1334 (BP · *R*_mw_)/IMT, where BP and *R*_mw_ are the BP values (systolic or diastolic) and midwall radius (systolic or diastolic), respectively. *R*_mw_ was calculated as (*R*_*e*_ − *R*_*i*_)/2, *R*_*e*_ and *R*_*i*_ being the external and internal radii, respectively. Systolic and diastolic *ε* were calculated as *R*_mw_/*R*_0_, where *R*_0_ is a reference value of midwall radius for an unstressed situation [17]. The EM was assessed assuming the linear elastic theory and the arterial wall as an isotropic homogeneous elastic material, according to the following equation [17, 18]: EM = 0.75(Δ*σ*/Δ*ε*), where Δ*σ*/Δ*ε* represents the ratio between the systolic-diastolic stress and systolic-diastolic strain. The EM was quantified for the CCA and CFA, because (according to the literature and our technical experience) the BA IMT could not be adequately visualized to ensure a high quality determination.

Third, a correlation analysis between arterial stiffness and BP levels was performed for both normal weight and obese groups. To this end, nonlinear and linear regression models for each group were obtained.

#### 2.3.4. Regional Arterial Stiffness

Pulse wave velocity (PWV, PWV = Δ*x*/Δ*t*) measurement (SphygmoCor 7.01, AtCor Medical, Sydney, Australia) allowed assessing aortic (carotid-femoral, cfPWV), lower-limb (femoral-pedal, fpPWV), and upper-limb (carotid-radial, crPWV) regional stiffness. The direct distance between the recording sites was considered as the pulse path length (Δ*x*) for the cfPWV. For the fpPWV, the pulse path length was the distance between the femoral and pedal recording sites, while for the crPWV the pulse path length was the distance between the suprasternal notch and the radial artery sampling site. The pulse transit time (Δ*t*) was defined obtaining the waves' foot-to-foot time delay (intersecting tangent algorithm), using the R wave of the ECG as a temporal reference. Real cfPWV was obtained multiplying the calculated cfPWV by a scaling factor of 0.8 [Figure 1(d)]. Correlation analysis between PWV and BP levels was performed for both normal weight and obese groups.

### 2.4. Statistical Analysis

Data analysis was performed using SPSS software (SPSS Inc., Illinois, USA). For data analysis, both normal weight and obese children were divided into age groups: 4–8, 8–12, and 12–15 years old. Values were expressed as mean ± standard deviation or prevalence (%, 95% confidence interval [CI]). Chi squared and Student's *t*-tests were used. A *p* value < 0.05 was considered significant. Linear regression models showed an adequate fit to BP-arterial stiffness relationships, statistically similar to those obtained using nonlinear models (exponential, logarithmic, and quadratic). Body weight-related differences in BP-arterial stiffness relationship were assessed by comparison of the models' slopes, analyzing the interaction between BP and anthropometric (normal weight or obesity) stage as covariables (Figure 3 and Table 3) [19]. A *p* value < 0.05 was considered significant.

## 3. Results

Table 1 shows cardiovascular risk factor prevalence and anthropometric, demographic, and hemodynamic characteristics for obese and normal weight children. When all the studied subjects were considered, there were no significant differences in sex distribution, age, or body height between obese and normal weight children. Similar results were obtained when the different age groups were analyzed.

Obese subjects aged twelve and older showed higher peripheral and central SBP and PP, with respect to children with normal weight. For all age groups, cPP (but not pPP) was higher in obese than in normal weight children (Table 1). There were no differences in DBP.

When all the subjects were considered, the prevalence of dyslipidemia and of diagnosed hypertension and/or hypertensive peripheral BP levels during the study was higher in obese than in normal weight children. Such differences varied depending on the age group analyzed. The prevalence of sedentary life styles was higher in obese children when all the subjects were considered (Table 1).

Table 2 shows arterial diameters and local and regional arterial stiffness parameters. When all the subjects were considered, obese children tend to show arterial diameters larger than those of the normal weight counterpart. The differences in arterial diameter would be explained mainly by the elder age group since obese children aged 12 and older showed larger CCA, CFA, and BA diameters with respect to normal weight children (statistically significant).

**Figure 2:**
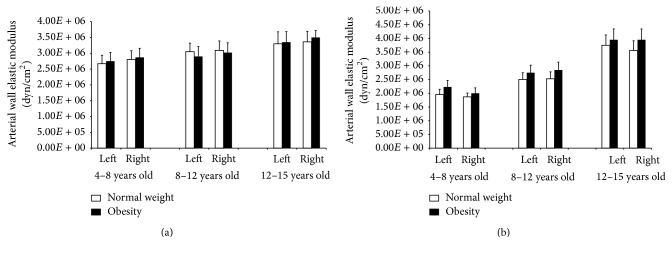
Common carotid (a) and femoral (b) arterial wall elastic modulus for normal weight and obese children and adolescents. There were no statistical differences between normal weight and obese arteries, when comparisons were done considering the same artery and hemibody (left or right), indicating that there were no significant intrinsic arterial wall elastic alterations associated with obesity.

In addition, with respect to those with normal weight, obese children aged 12 and older showed lower carotid and femoral distensibility (higher local arterial stiffness) (*p* < 0.05). There were no differences in BA distensibility (stiffness) between normal weight and obese children. When local arterial stiffness values were normalized for BP by calculating the *β* Index, there were no differences between normal weight and obese children, with independence of the arterial segment considered (Table 2). Furthermore, there were no differences in the CCA and CFA elastic modulus between normal weight and obese children (Figure 2).

When regional arterial stiffness parameters were considered (PWV), there were no differences between normal weight and obese children (Table 2), with independence of the arterial pathway considered.

**Figure 3:**
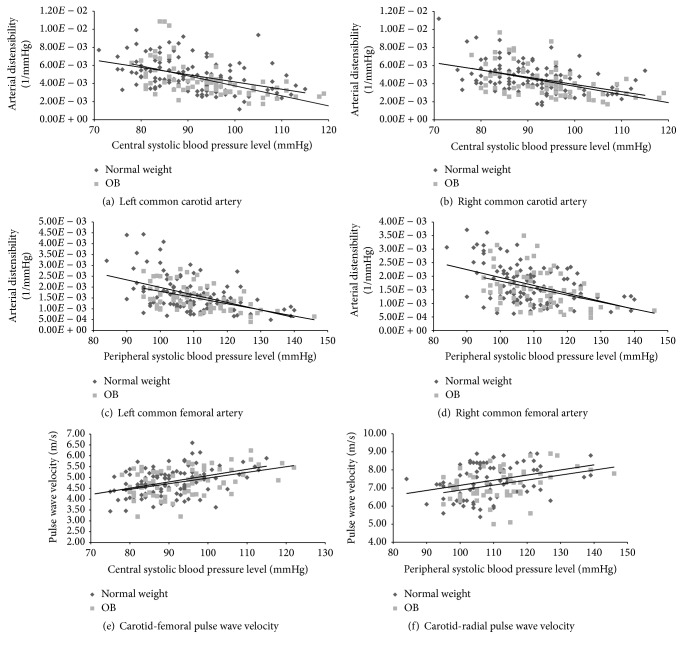
Correlation analysis (linear regression plots) between stiffness parameters and blood pressure levels for normal weight (NW) and obese (OB) children and adolescents. Nonstatistical differences were found when equations (for the same artery) were compared (slopes comparisons) between NW and OB groups (data shown in Table 3), indicating a similar arterial stiffness-blood pressure association (relationship).

Left and right CCA and CFA (but not BA) distensibility as well as cfPWV and crPWV (but not fpPWV) showed a negative and statistically significant correlation with BP levels (Figure 3 and Table 3). The models for normal weight and obese children and adolescents were similar, suggesting that the reduced distensibility observed in obese children could be explained by a BP-dependent effect and not by changes in the arterial stiffness-BP physiological relationship.

## 4. Discussion

To our knowledge, this study provides for the first time data related to arterial changes associated with obesity in asymptomatic children and adolescents, analyzing different vascular territories and the role of arterial BP. The work's main results were as follows:

- Hemodynamic and vascular differences between normal weight and obese subjects were mainly observed in the older-age group (subjects aged 12 and older). Compared with normal weight subjects, obese adolescents aged twelve and older had not only higher peripheral BP levels but also higher central (aortic) BP levels.
- Carotid and femoral distensibility, but not brachial artery distensibility, were decreased in obese adolescents aged 12 and older. Therefore, the arterial stiffness increase associated with obesity would be age- and arterial segment-dependent.
- When stiffness values were normalized for arterial BP (*β* Index), there were no differences in arterial distensibility between obese and normal weight subjects. Similar results were observed when the elastic modulus (a true evaluator of the wall artery material stiffness regardless of its geometry and/or size) of carotid and femoral arteries was compared between obese and normal weight subjects (Figure 2). Moreover, the local and regional arterial stiffness-BP relationships were similar between obese and normal weight subjects (Figure 3 and Table 3), reinforcing that the observed differences in arterial stiffness could be explained by differences in BP levels. Then, at least in theory, these results could indicate that the increased arterial stiffness in obese subjects would not be explained by intrinsic changes in the arterial wall or by changes in the arterial stiffness-BP physiological relationship.
- There were no differences in regional stiffness between normal weight and obese children, which might be explained by the opposite effects on stiffness (PWV) associated with the hemodynamic (i.e., BP) and geometrical (i.e., diameters) changes observed in obese children.

### 4.1. Peripheral and Central Blood Pressure

Compared with normal weight subjects, the obese adolescents aged 12 and older showed similar diastolic BP levels, but higher central and peripheral SBP and PP. These findings are in agreement with previous works in which young normotensive patients were studied [20]. The hemodynamic differences observed have been proposed to be on the basis of target organ damage development (i.e., left ventricular hypertrophy) [21, 22]. The association between the rising prevalence of obesity in the pediatric population and BP increase has been described. Furthermore, it has been shown that BP increases with the increasing BMI [23] and elevated cSBP and cPP could be associated with increased cardiovascular risk [24, 25]. Looking at our and other works' findings, it could be said that childhood obesity and its association with central and peripheral BP rise may accelerate the aging process and predispose to an increase in cardiovascular risk.

### 4.2. Arterial Diameters and Arterial Stiffness

Carotid, femoral, and brachial diameters were larger in obese adolescents aged 12 and older than in normal weight adolescents from the same age group. These results could be explained by the increase in BP that would lead to a passive distension of the arterial tree. In relation with this, it has been suggested that childhood obesity may lead to hemodynamic changes, with an increased systemic blood flow that responds to changes in the ventricular function, as an adaptative mechanism that could contribute to explaining the increase in the arterial working diameters [3, 26, 27]. In relation to this, in a recent work of our group in which noninvasive methods were employed, obese children and adolescents showed increased left ventricle stroke volume and cardiac output [28].

Obese subjects aged 12 and older showed lower carotid and femoral distensibility [Table 2]. In contrast, there were no differences in brachial artery distensibility between obese and normal weight subjects, with independence of the age considered. Looking at our results, it could be said that the impact of childhood obesity in the arterial system is heterogeneous (dissimilar impact in different vascular territories) and obesity-related vascular changes may be age-dependent. In agreement with this, Charakida et al., studying vascular phenotypes related to childhood obesity, did not find differences between obese and normal weight prepubertal children, but they described arterial changes in obese children aged 10 and older. The authors suggested that obese children under 10 years of age might have ongoing compensatory mechanisms to adapt to the consequences of obesity and adiposity over the arterial tree. Those compensatory mechanisms might become insufficient in children aged 10 and older [26]. Our findings might be explained, at least partially, by this hypothesis. Then, we could say that the metabolic disturbances that might be present in childhood obesity could have early deleterious vascular effects. However, the vascular changes would become “evident” (in terms of arterial stiffness changes) in subjects aged 12 and older, when compensatory mechanisms are not enough [8, 26].

It is known that some of the named metabolic disturbances in childhood obesity are related to increased insulin blood levels and insulin resistance. Puberty has been studied as related with a decrease in insulin resistance, which could contribute to explaining our findings [29, 30]. In contrast, some authors found that this state is associated with an increase in vascular distensibility in obese subjects and in those with type 2 diabetes mellitus, arguing that this could lead to an early maturation of vascular system [31]. In a longitudinal study of children of 5 to 19 years of age by McGavock et al. [32], they found that obese children had elevated SBP. Furthermore, weight gain was associated with an age-related rise in BP and also linked to changes in cardiac output, without any change in arterial compliance. The authors suggested that changes in arterial stiffness might be related to the duration of overweight or the degree of obesity.

As it is known, arterial stiffness depends on structural components of the arterial wall, BP levels, and smooth muscle tone. In our study, while analyzing the relationship between the differences in arterial stiffness in both carotid and femoral arteries in obese children aged 12 and older, we found that once stiffness values were normalized for BP levels (*β* Index) there were no differences between obese and normal weight subjects. A conceptually similar result was observed when the elastic modulus of the arterial wall was analyzed. About this, a similar elastic modulus suggests that obese and normal weight subjects have similar arterial wall intrinsic elastic properties. Then, it could be said that, at least in early stages of life, obesity would not be associated with changes in the arterial wall intrinsic stiffness (i.e., wall components), but with BP-dependent stiffness changes. Finally, and supporting this data, when the arterial stiffness-BP relationships were analyzed, similar associations were found for normal weight and obese subjects. Then, arteries from both groups would be “working” following a similar arterial wall stiffness-BP relationship. Furthermore, as was previously discussed for animal and human studies, the finding of similar stiffness-BP relationships suggests there are no differences in the smooth muscle tone level (determinant of the arterial stiffness) and/or significant structural changes between arteries from obese and normal weight subjects [17, 18, 33].

At least in theory, the excess of adipose tissue, which leads to an increased volume in the vascular system and to an inflammatory state, may contribute to initiating the vascular remodelling in which the high BP levels may have a key role. Taking into account that stated above, the increased arterial stiffness observed in obese children and adolescents could be reversed controlling BP levels [34]. This, in turn, could be achieved (at least partially) by body weight control.

When the regional arterial stiffness was analyzed, the results showed that there were no differences in PWV between obese and normal weight subjects. Although this finding could be thought of as paradoxical, since PWV and BP levels showed a positive (statistically significant) relationship and obese children showed higher BP levels, the result could be explained by the Moens-Korteweg equation. This equation allows analyzing hemodynamic and arterial determinants of the PWV [33]: PWV^2^ = EM · *h*/2 · *D* · *ρ*, where EM is the arterial wall elastic modulus (that is to say, the “intrinsic wall stiffness”), *h* is the arterial wall thickness, *D* is arterial diameter, and *ρ* is blood density. This way, we could hypothesize that the obesity-associated changes would act in contrary ways resulting in no significant changes in PWV.

### 4.3. Clinical Connotations

As it is well known, arterial stiffness is related to increased ventricular afterload, lower cardiac systodiastolic function, impaired vascular functional capability (i.e., ability to cushion or buffer vascular pulsatility), and increased cardiovascular risk. Looking at our findings, it could be said that childhood obesity is associated with age-related arterial changes (in some vascular pathways) that could lead to cardiovascular damage and risk increase. Furthermore, this work contributes to the proposal that obesity-associated changes may progress early in life becoming evident in older children. In addition, our work gives data linking obesity-associated BP increase and vascular changes. Then, interventions aimed at controlling BP (including weight control) would result in an improvement in vascular properties.

## 5. Conclusions

Childhood obesity was associated with age-dependent increase in local carotid and femoral, but not brachial, arterial stiffness, which became significant in subjects aged 12 and older. Stiffness changes were not associated with intrinsic changes in the arterial wall elasticity but could be explained by BP-dependent passive arterial distension determined by the increased peripheral and central aortic BP levels observed in obese subjects.

## Figures and Tables

**Table 1:** Anthropometrical and hemodynamic characteristics and prevalence of cardiovascular risk factors.

	Total			4–8 years old			8–12 years old			12–15 years old		
	Normal weight	Obesity	*p*	Normal weight	Obesity	*p*	Normal weight	Obesity	*p*	Normal weight	Obesity	*p*
Number of subjects (% female)	137 (39)	84 (41)	0.876	25 (40)	18 (50)	0.765	48 (40)	40 (48)	0.745	57 (40)	21 (33)	0.476
Age (years)	10.9 ± 2.8	10.6 ± 2.7	0.465	6.7 ± 0.9	6.5 ± 0,8	0.389	10.5 ± 1.2	10.3 ± 1.2	0.265	13.7 ± 0.8	13.4 ± 1.0	0.111
Body height (m)	1.42 ± 0.2	1.41 ± 0.2	0.502	1.22 ± 0.1	1.23 ± 0.1	0.555	1.41 ± 0.1	1.40 ± 0.1	0.359	1.62 ± 0.1	1.63 ± 0.1	0.234
Body weight (Kg)	38.6 ± 13.4	58.6 ± 21.3	**<0.001**	22.1 ± 2.5	34.0 ± 5.7	**<0.001**	34.4 ± 6.9	54.6 ± 12.6	**<0.001**	50.9 ± 8.8	83.7 ± 16.0	**<0.001**
Body mass index (kg/m^2^)	18.0 ± 2.2	26.8 ± 4.8	**<0.001**	15.3 ± 0.8	22.0 ± 2.9	**<0.001**	17.5 ± 1.5	26.4 ± 3.4	**<0.001**	19.8 ± 1.7	31.1 ± 5.1	**<0.001**

Heart rate (b.p.m.)	82 ± 14	79 ± 13	0.596	87 ± 10	89 ± 21	0.715	82 ± 17	78 ± 11	0.165	76 ± 11	76 ± 14	0.965
Peripheral SBP (mmHg)	108 ± 11	113 ± 11	**0.008**	101 ± 9	106 ± 7	0.074	109 ± 9	109 ± 7	0.863	111 ± 10	119 ± 12	**0.012**
Peripheral DBP (mmHg)	60 ± 8	60 ± 7	0.845	58 ± 7	63 ± 11	0.984	60 ± 6	58 ± 6	0.131	59 ± 8	62 ± 10	0.112
Peripheral PP (mmHg)	48 ± 10	52 ± 9	**0.006**	43 ± 8	46 ± 10	0.228	48 ± 10	51 ± 7	0.118	52 ± 9	58 ± 8	**0.008**

Central (aortic) SBP (mmHg)	91 ± 9	94 ± 10	**0.002**	84 ± 8	88 ± 7	0.091	92 ± 8	92 ± 7	0.875	94 ± 9	104 ± 10	**<0.001**
Central (aortic) DBP (mmHg)	62 ± 8	61 ± 7	0.932	60 ± 8	60 ± 5	0.866	63 ± 8	59 ± 7	0.056	62 ± 7	65 ± 8	0.103
Central (aortic) PP (mmHg)	29 ± 7	33 ± 8	**<0.001**	24 ± 6	29 ± 8	**0.037**	29 ± 7	33 ± 6	**0.010**	32 ± 7	39 ± 9	**<0.001**

Hypertension (%, CI 95%)	10 (6–16)	32 (22–42)	**<0.001**	12 (0–25)	39 (16–61)	**0.039**	17 (6–27)	25 (12–38)	0.923	7 (0–14)	43 (22–43)	**<0.001**
Dyslipidemia (%, CI 95%)	2 (0–4)	16 (8–23)	**<0.001**	8 (0–19)	6 (0–16)	0.756	2 (0–6)	20 (8–32)	**0.029**	0 (0-0)	10 (0–22)	**0.018**
Sedentarism (%, CI 95%)	46 (38–55)	66 (54–76)	**0.005**	68 (50–86)	67 (45–88)	0.926	44 (30–58)	70 (56–84)	0.583	40 (28–53)	57 (36–78)	0.186

Values expressed as mean value ± standard deviation or percentage (prevalence) and confidence interval (95%). SBP, DBP, and PP: systolic, diastolic, and pulse blood pressure, respectively. Hypertension: diagnosed hypertension and/or hypertensive levels during study. A *p* < 0.05 was accepted as statistically significant.

**Table 2:** Carotid, femoral, and brachial arteries “local” stiffness and aortic, upper-limb, and lower-limb “regional” stiffness.

	Total			4–8 years old			8–12 years old			12–15 years old		
	Normal weight	Obesity	*p*	Normal weight	Obesity	*p*	Normal weight	Obesity	*p*	Normal weight	Obesity	*p*
Systolic diameter left CCA (mm)	6.3 ± 0.6	6.5 ± 0.7	**0.004**	5.9 ± 0.5	6.0 ± 0.6	0.349	6.3 ± 0.6	6.5 ± 0.5	0.092	6.4 ± 0.7	7.0 ± 0.4	**0.001**
Diastolic diameter left CCA (mm)	5.5 ± 0.6	5.8 ± 0.6	**0.004**	5.2 ± 0.5	5.3 ± 0.5	0.281	5.6 ± 0.6	5.8 ± 0.5	0.140	5.6 ± 0.7	6.2 ± 0.4	**<0.001**
Distensibility left CCA (1/mmHg × 10^−3^)	4.9 ± 1.9	4.2 ± 1.8	**0.011**	6.2 ± 1.9	5.4 ± 2.2	0.211	4.9 ± 1.5	4.5 ± 1.9	0.317	4.3 ± 1.5	3.3 ± 0.7	**0.004**
*β* Index left CCA	3.2 ± 1.5	3.5 ± 1.1	0.118	2.5 ± 0.7	3.1 ± 1.6	0.104	3.0 ± 1.1	3.4 ± 1.1	0.095	3.5 ± 1.4	3.8 ± 0.8	0.325

Systolic diameter right CCA (mm)	6.5 ± 0.7	6.7 ± 0.7	0.109	6.1 ± 0.7	6.1 ± 0.5	0.984	6.5 ± 0.6	6.7 ± 0.5	0.086	6.8 ± 0.6	7.2 ± 0.4	**0.006**
Diastolic diameter right CCA (mm)	5.8 ± 0.6	5.9 ± 0.7	0.154	5.4 ± 0.7	5.4 ± 0.4	0.965	5.8 ± 0.6	5.9 ± 0.5	0.149	6.0 ± 0.5	6.4 ± 0.4	**0.009**
Distensibility right CCA (1/mmHg × 10^−3^)	4.7 ± 1.6	4.2 ± 1.7	**0.043**	5.9 ± 2.0	5.4 ± 2.3	0.478	4.8 ± 1.4	4.3 ± 1.5	0.131	3.9 ± 1.3	3.2 ± 0.8	**0.023**
*β* Index right CCA	3.2 ± 1.1	3.6 ± 1.2	0.051	2.6 ± 0.8	3.0 ± 1.4	0.242	3.1 ± 1.1	3.6 ± 1.2	0.062	3.7 ± 1.3	4.1 ± 1.3	0.274

Systolic diameter left CFA (mm)	6.0 ± 1.0	6.4 ± 1.0	**0.004**	5.0 ± 0.5	5.6 ± 0.6	**0.002**	5.9 ± 0.7	6.3 ± 0.7	**0.009**	6.8 ± 1.0	7.5 ± 0.9	**0.006**
Diastolic diameter left CFA (mm)	5.6 ± 1.0	6.0 ± 1.0	**0.005**	4.6 ± 0.5	5.2 ± 0.6	**0.001**	5.4 ± 0.7	5.8 ± 0.7	**0.024**	6.3 ± 1.0	7.1 ± 7.1	**0.005**
Distensibility left CFA (1/mmHg × 10^−3^)	1.8 ± 1.2	1.6 ± 0.8	**0.047**	2.3 ± 0.9	1.9 ± 0.8	0.113	1.9 ± 1.5	1.7 ± 0.8	0.719	1.4 ± 0.7	1.1 ± 0.4	**0.049**
*β* Index left CFA	8.7 ± 3.9	9.4 ± 4.0	0.212	6.5 ± 2.6	7.6 ± 2.5	0.185	8.7 ± 4.1	8.4 ± 3.2	0.734	10.3 ± 3.7	11.9 ± 4.5	0.317

Systolic diameter right CFA (mm)	5.9 ± 1.0	6.3 ± 1.3	**0.027**	5.0 ± 0.4	5.4 ± 0.7	**0.014**	5.8 ± 0.7	6.3 ± 0.7	**0.002**	6.7 ± 0.9	7.5 ± 0.9	**0.002**
Diastolic diameter right CFA (mm)	5.5 ± 1.0	5.9 ± 1.2	**0.022**	4.6 ± 0.4	5.0 ± 0.6	**0.012**	5.4 ± 0.7	5.9 ± 0.7	**0.003**	6.2 ± 0.9	7.0 ± 0.8	**0.001**
Distensibility right CFA (1/mmHg × 10^−3^)	1.8 ± 1.0	1.5 ± 0.7	**0.029**	2.3 ± 0.8	2.1 ± 1.0	0.487	1.8 ± 1.3	1.6 ± 0.6	0.323	1.5 ± 0.7	1.1 ± 0.4	**0.049**
*β* Index right CFA	8.3 ± 3.8	9.4 ± 4.1	0.076	6.7 ± 3.9	7.9 ± 4.5	0.401	8.5 ± 3.8	9.1 ± 3.3	0.477	9.8 ± 3.8	11.4 ± 4.7	0.164

Systolic diameter BA (mm)	2.9 ± 0.5	3.2 ± 0.5	**0.014**	2.5 ± 0.3	2.8 ± 0.3	**0.041**	2.9 ± 0.5	3.1 ± 0.4	0.156	3.2 ± 0.3	3.6 ± 0.4	**0.013**
Diastolic diameter BA (mm)	2.8 ± 0.5	3.0 ± 0.5	**0.024**	2.4 ± 0.2	2.7 ± 0.4	**0.042**	2.7 ± 0.5	2.9 ± 0.3	0.221	3.0 ± 0.4	3.5 ± 0.4	**0.016**
Distensibility BA (1/mmHg × 10^−3^)	1.5 ± 1.2	1.4 ± 0.8	0.547	1.7 ± 1.2	1.6 ± 0.8	0.914	1.6 ± 1.5	1.4 ± 1.0	0.719	1.4 ± 0.9	1.0 ± 0.3	0.241
*β* Index BA	12.8 ± 7.9	12.1 ± 6.8	0.726	11.4 ± 6.8	9.4 ± 3.8	0.500	13.1 ± 8.6	12.2 ± 7.6	0.755	13.3 ± 8.7	13.1 ± 6.1	0.947

Carotid-femoral PWV real (m/s)	4.7 ± 1.4	4.8 ± 0.7	0.401	4.4 ± 0.6	4.6 ± 0.7	0.504	4.7 ± 0.6	4.7 ± 0.5	0.983	5.1 ± 0.6	5.3 ± 0.5	0.363
Femoropedal PWV (m/s)	7.0 ± 1.1	7.1 ± 1.3	0.662	7.1 ± 0.9	7.3 ± 1.5	0.774	6.7 ± 0.8	6.7 ± 1.4	0.992	7.0 ± 1.3	7.8 ± 0.9	0.070
Carotid-radial PWV (m/s)	7.6 ± 1.2	7.4 ± 1.3	0.283	7.6 ± 1.2	6.8 ± 1.1	0.075	7.3 ± 0.9	6.7 ± 0.7	0.074	7.6 ± 1.0	7.8 ± 0.6	0.317

Values expressed as mean ± standard deviation. CCA: common carotid artery. CFA: common femoral artery. BA: brachial artery. PWV: pulse wave velocity. A *p* < 0.05 was accepted as statistically significant.

**Table 3:** Correlation analysis between stiffness parameters and blood pressure levels.

	Normal weight				Obesity				*p* (slope differences)
	Model	*R*	*p*	95% CI	Model	*R*	*p*	95% CI	
Left common carotid artery distensibility	−8.06**E** − 5**x** + 0.012	0.428	<0.001	−1,12*E* − 04–−5,00*E* − 05	−1.10**E** − 4**x** + 0.015	0.554	<0.001	−1,49*E* − 04–−7,2*E* − 05	0.232
Right common carotid artery distensibility	−8.01**E** − 5**x** + 0.012	0.437	<0.001	−1,11*E* − 04–−4,9*E* − 05	−9.03**E** − 5**x** + 0.013	0.496	<0.001	−1,28*E* − 04–−5,3*E* − 05	0.672
Left common femoral artery distensibility	−3.47**E** − 5**x** + 0.005	0.467	<0.001	−4,80*E* − 05–−2,2*E* − 05	−2.85**E** − 5**x** + 0.005	0.518	<0.001	−4,00*E* − 05–−1,7*E* − 05	0.502
Right common femoral artery distensibility	−2.92**E** − 5**x** + 0.005	0.448	<0.001	−4,10*E* − 05–1,80*E* − 05	−2.57**E** − 5**x** + 0.004	0.399	<0.001	−4,00*E* − 05–−1,2*E* − 05	0.700
Carotid-femoral pulse wave velocity	0.029**x** + 2.177	0.398	<0.001	0.016–0.042	0.026**x** + 2.369	0.433	<0.001	0.013–0.039	0.744
Carotid-radial pulse wave velocity	0.028**x** + 4.318	0.318	<0.001	0.015–0.040	0.027**x** + 4.111	0.359	<0.001	0.011–0.035	0.756

Analysis between arterial stiffness parameters (*y*-axis) and systolic blood pressure levels (*x*-axis). CI: confidence interval. All regressions reached statistical significance (*p* < 0.001). There were no differences in the slopes (model comparison) between normal weight and obese children. A *p* < 0.05 was accepted as statistically significant.
